# Guidelines for selecting variability measure in limited-size ANOVA experiments

**DOI:** 10.1038/s41598-026-54181-0

**Published:** 2026-06-08

**Authors:** Mara Gabbrielli, Elena Valkama, Roberta Calone, Simone Bregaglio, Luisa Manici, Giorgio Ragaglini, Alessia Perego, Marco Acutis

**Affiliations:** 1 Department of Agricultural and Environmental Sciences, University of Milan, Via Celoria 2, Milan, 20133 Italy; 2 Unit of Bioeconomy and Environment, Sustainability Food Systems and Society, Natural Resources Institute Finland (Luke), Kulttuurikuja 1, Turku, 20800 Finland; 3 Consiglio per la ricerca in agricoltura e l’analisi dell’economia agraria (CREA), Centro di ricerca Orticoltura e Florovivaismo, Corso degli Inglesi 508, Sanremo, 18038 Italy; 4 Consiglio per la ricerca in agricoltura e l’analisi dell’economia agraria (CREA), Centro di ricerca Agricoltura e Ambiente, Via di Corticella 133, Bologna, 40128 Italy

**Keywords:** Standard deviation reporting, Small sample size, Pooled standard deviation, Variance heterogeneity, Biological techniques, Computational biology and bioinformatics, Ecology, Ecology, Zoology

## Abstract

Graphical representation of variability is essential for accurately communicating scientific results in applied sciences. In ANOVA experiments with a low number of replicates (n), the selection between pooled or individual standard deviations (SDs) poses a methodological challenge. The aim of this study is to offer practical guidelines to help researchers decide whether to use pooled or individual SDs in tables and figures, when the number of replicates is low. This study uses extensive Monte Carlo simulations (over 2,000 scenarios) to investigate the distributional behaviour of SD estimates across different replications and heterogeneity levels. We compare the performance of pooled versus individual SDs using the Mean Absolute Deviation (MAD) from the true population values, and we evaluate the utility of Levene’s and F_max_ (Hartley’s) tests in guiding this choice. Results show that pooled SDs offer superior accuracy under homogeneity or low heterogeneity conditions, particularly with *n* ≤ 4, while individual SDs are preferable when variance heterogeneity is moderate to high and the replication number is higher (*n* ≥ 5). Levene’s test generally outperforms the F_max_ test in supporting the correct selection of the variability measure, especially in multi-group settings. The guidelines proposed can be directly applied to many experimental settings in applied sciences, where the number of replicates and treatments is often limited.

**Supplementary Information:**

The online version contains supplementary material available at 10.1038/s41598-026-54181-0.

## Introduction

Representing variability associated with observed mean values is essential for accurately communicating scientific results and extracting treatment variability for meta-analyses. Variability not only informs readers about the spread of data but also reflects the precision of the estimated mean^1^. Commonly used indicators of variability are standard deviation (SD), standard error (SE), and confidence interval (CI). Each measure serves a distinct purpose, and the choice depends on the specific context. As sample size usually means the number of observational units within a sample or group, often denoted as (n), henceforth, “sample size” will refer to the number of replicates. Standard Deviation is a descriptive statistic that quantifies the spread of individual data points around their mean, making it a general measure of variability. Standard Error is an inferential statistic derived from SD and sample size and represents the variability of the sample mean, providing insight into how much the sample mean might differ from the population mean as a function of the number of samples taken. Confidence Intervals extend the SE by providing a range of values that has a specified probability (level of confidence, e.g., 95%) of containing the true population mean. Like SE, CI is influenced by both SD and the sample size.

Despite their distinct definitions and intended uses, these measures are not always used consistently in practice and may sometimes be applied interchangeably. Scientific journals often have specific guidelines for reporting measures of variability, but many leave the choice to the authors. For instance, Krzywinski and Altman^2^ reported that in Nature Methods, error bars were based on SD in 45% of articles, SE in 49%, and CI in only 1%. Tang et al.^3^ observed similar trends in biomedical research. In both studies^2,3^, the distinction between pooled and individual-group SDs appears to have been overlooked.

There is no consensus among scientists on the “best” measure of variability. While Tang et al.^3^ advocate for SE, Patience et al.^4^ favour CI, and Barde and Barde^5^, as well as Andrade^6^, support the use of SD. Furthermore, Barde and Barde^5^ state that the use of SE should be limited to the construction of CI. Despite these differences, all of these authors agree on the importance of reporting sample size alongside measures of variability and explaining the rationale of the chosen metric. However, only a few studies have addressed the representation of variability in experiments with small sample sizes (e.g., 2–5 replicates per treatment), which is common in applied sciences, for example in agro-environmental and soil science research, as well as in medical and animal sciences.

In such small-scale experiments, the choice of the variability measure becomes particularly challenging. Cummings et al.^7^ recommend plotting individual data points rather than relying on error bars when sample size is extremely small (e.g., *n* = 3). Webster^8^ suggested using a single SE calculated from the pooled within-group mean square of ANOVA for balanced experiments, as it provides a consistent measure across treatments. Webster also proposed replacing pooled error bars with the Least Significant Difference (LSD) value, which can be converted to pooled SD using exact methods^9^.

While the choice between pooled and group-specific SDs is inherently a descriptive issue, it is methodologically relevant in the context of ANOVA-based experimental designs, as the way variability is summarised and reported can influence the communication of inferential results. Although explicit references advocating pooled statistics for representing variability are limited, their use is justified when data are analysed with ANOVA. The pooled Mean Square Error (MSE) underpins ANOVA, as it provides the estimate of within-group variability. The assumption of homogeneity of variance is therefore critical, since it justifies pooling the variances from different groups into a single, shared measure. Once homogeneity of variance has been verified and ANOVA conducted, using a pooled measure of variability across all means is consistent with the applied statistical framework. This approach, based on pooled variances, offers several advantages: it mitigates the bias of SD associated with small sample sizes, reduces the width of CIs, and ensures coherence with post-hoc tests like LSD^10^. However, these benefits depend on the validity of the variance homogeneity assumption. When this assumption is not satisfied, alternative analytical approaches should be considered, including Welch’s ANOVA, heteroscedasticity-robust or mixed-effects models, Bayesian hierarchical frameworks, and non-parametric methods, which provide more reliable inference under such conditions. In the presence of variance heterogeneity, pooled variability measures can lead to misleading interpretations, particularly for groups with larger variances.

The aims of this study are: (i) to provide guidance on the distribution of SD for small to medium sample sizes (*n* = 2–10), which, although established for decades^11^, appears to have been largely overlooked in the presentation of data variability; (ii) to determine whether tests for homogeneity of variances can inform the selection of a variability measure, thereby guiding the choice between using single-group (individual) or pooled SDs; (iii) to offer criteria for choosing between pooled or individual SDs when presenting means and SDs in tables and graphs for limited-size ANOVA experiments.

## Methods

### Simulations setup

**Fig. 1:**
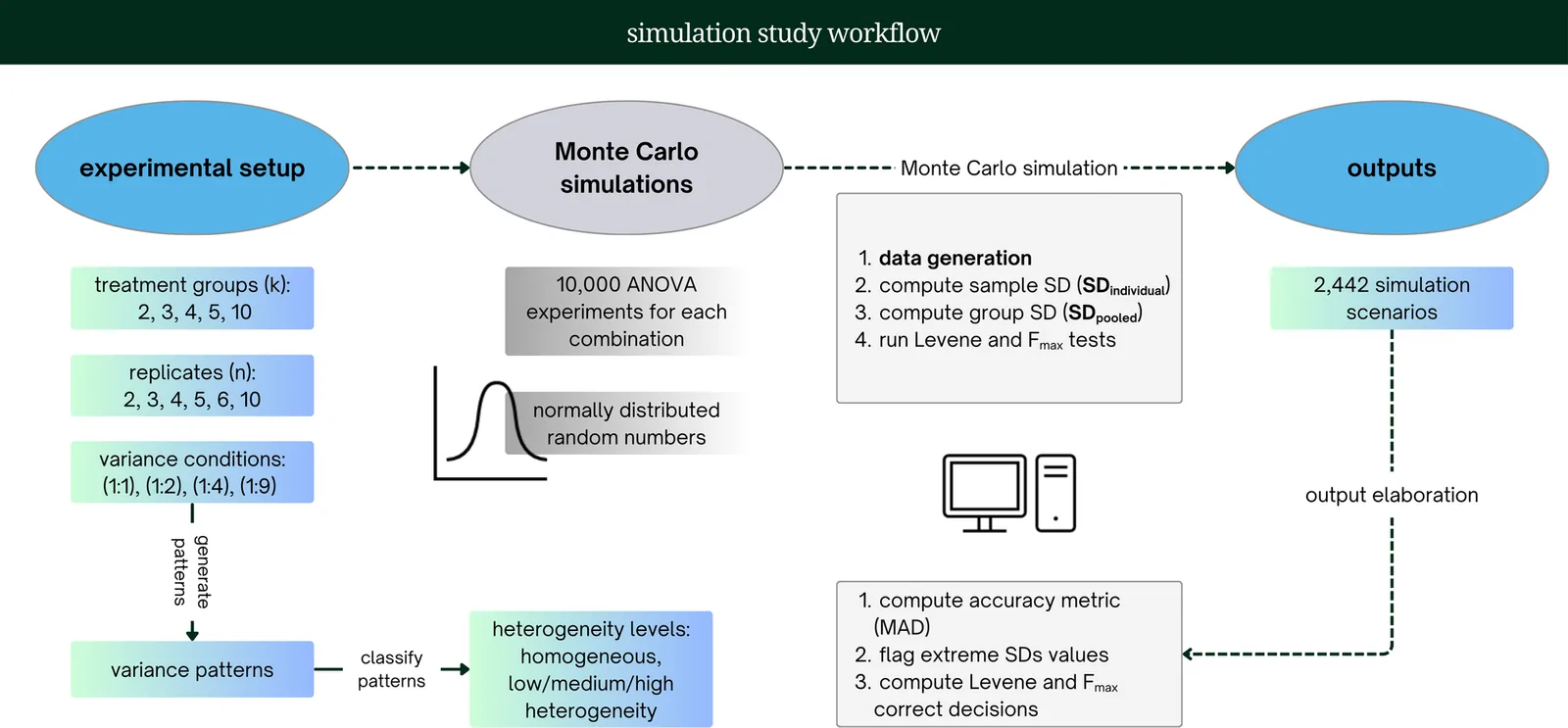
Overview of the study workflow. The diagram illustrates the experimental setup and Monte Carlo simulation setups, the main steps of each simulation, the total number of simulated scenarios, and the procedures for output processing.

This study is based on Monte Carlo simulations designed to evaluate the behaviour of SD estimates and variance homogeneity tests in small-sample ANOVA settings (Fig. 1). Here, two types of SD estimates are considered: the standard deviation of each treatment level (SD_individual_), and the root mean squared error within treatments (SD_pooled_, Eq. 1) defined as the square root of the within-treatment mean square error (MSE). The MSE is computed as the sum of squared errors (SSE) divided by the within-group degrees of freedom (*N − k*, where *N* is the total number of observations and *k* is the number of treatments means), corresponding to the unbiased estimator of the common within-group variance. SSE is the double summation of squared deviations of each observation *x*_*ij*_ from its corresponding group mean $\:{\stackrel{\prime }{X}}_{j}$, where *i* indexes observations within group *j*, and *n*_*j*_ is the sample size of group *j*. Thus, the pooled standard deviation represents the square root of the average within-group variability across all treatments.

1 $$\:{SD}_{pooled}=\sqrt{MSE}=\sqrt{\frac{SSE}{N-k}}=\sqrt{\frac{\sum\:_{j=1}^{k}\sum\:_{i=1}^{{n}_{j}}{\left({x}_{ij}-{\stackrel{-}{X}}_{j}\right)}^{2}}{N-k}}$$

All simulations were based on 10,000 experiments^12^ generated using normally distributed random numbers. Normal deviates were produced using the *np.random.normal* function from the Python Library NumPy, which employs the PCG64 pseudo-random number generator^13^ and the Ziggurat transformation algorithm^14^. This combination ensures high-quality sampling that conforms to theoretical distributional properties, as confirmed by recent assessments^15^.

To assess the performance of different methods for selecting variability measure, and to evaluate whether homogeneity tests can inform the choice between SD_individual_ and SD_pooled_, ANOVA experiments were simulated with 2, 3, 4, 5, and 10 treatment groups (k). Each group was assigned an equal mean (set to 10) and varying replication levels (2, 3, 4, 5, 6, or 10). Sample size was thus classified according to the number of replicates (n) per treatment group as follows: very small (2 ≤ *n* ≤ 3), small (4 ≤ *n* ≤ 5) and medium (6 ≤ *n* ≤ 10). For each of the resulting 30 combinations, four variance ratios were generated: 1:1 (homogeneous variances), 1:2, 1:4, and 1:9 (heterogeneous variances), corresponding to standard deviation ratios between the largest SD to smallest SD of approximately 1, 1.41, 2, and 3 (Supplementary Fig. S1). These ratios coincide with the values reported in literature in studies examining variance ratios^10,16–18^. It is worth noting that, in real-world ANOVA experiments, homogeneous variances are either assumed a priori based on the researcher’s expert opinion or evaluated through variance homogeneity tests and/or residual plots; however, in small sample-size experiments, graphical assessment is not feasible due to the limited number of residuals.

To systematically explore possible patterns of variance heterogeneity, all unique patterns of group-level variances were generated for each group size using a combinatorial approach. Specifically, the *combinations_with_replacement* function from the Python Library Reference^19^ was used to enumerate all unordered combinations with repeated elements drawn from a predefined set of variance levels (1, 2, 4, 9). This method ensures complete and non-redundant coverage of all possible variance configurations, as it excludes permutations of identical values that would otherwise result in statistically equivalent scenarios.

For example, in the case of three groups (k = 3), the full set of 20 unique patterns includes four homogeneous configurations and 16 heterogeneous combinations (see Supplementary Fig. S2 for details):

(i) homogeneous:
  (1,1,1), (2,2,2), (4,4,4), (9,9,9);
(ii) heterogeneous:
  (1,1,2), (1,1,4), (1,1,9),
  (1,2,2), (1,2,4), (1,2,9),
  (1,4,4), (1,4,9),
  (1,9,9), (2,2,4), (2,2,9),
  (2,4,4), (2,4,9),
  (2,9,9), (4,4,9), (4,9,9).

These configurations capture all possible relative variance structures among three groups under the specified variance levels. The same procedure was applied to the five group sizes (k = 2, 3, 4, 5, 10), each evaluated under six replication levels (2, 3, 4, 5, 6, and 10 replicates), yielding a total of 2,442 unique simulation scenarios. The simulation grid comprises 10 patterns for k = 2, 20 for k = 3, 35 for k = 4, 56 for k = 5, and 286 for k = 10. Each pattern, including the homogeneous configuration, was combined with all replication levels, to ensure comparability across experimental conditions.

To support interpretation and generalization of the results, all variance patterns were normalized and classified according to their level of heterogeneity. Normalization was performed by dividing each variance value in a pattern by the smallest variance within that pattern. This yields a scale-invariant representation that emphasizes relative differences in variability rather than absolute magnitudes. For example, the patterns (1, 2, 2), (2, 4, 4), and (5, 10, 10) all normalize to the same structure (1.0, 2.0, 2.0), indicating that they are functionally equivalent in terms of relative heterogeneity, despite differing in absolute variance values. After normalization, the degree of heterogeneity was quantified using the coefficient of variation (CV) of the SDs (i.e., the square roots of the variances). This metric captures the dispersion of variability across groups in a scale-independent manner. Based on the distribution of CV values within each group size (k), patterns were classified into four heterogeneity levels (Supplementary Fig. S2):

(i) homogeneous: all variances equal (CV = 0);
(ii) low heterogeneity: CV values in the lower third of the CVs distribution for the same k;
(iii) medium heterogeneity: CV values in the middle third;
(iv) high heterogeneity: CV values in the upper third.

These thresholds were calculated separately for each group size to account for differences in the range and structure of possible variance patterns. In the case of k = 2, no patterns fall into the low heterogeneity level. The resulting classification supports structured comparisons of method performance under increasingly heterogeneous variance conditions, representing a practical approach to present the data in a synthetic manner, although it is not grounded in a probabilistic framework.

Results are based on 10,000 simulations per condition. Visual representation of data was carried out using R^20^, which was used to produce figures and summarise the main results.

### Metrics for evaluating the comparative performance of pooled and individual SDs

#### Accuracy of variability representation

To evaluate the accuracy of SD representation, the Mean Absolute Deviation (MAD) was used. For each simulation, at the individual group level and for the pooled estimate across all groups, we calculated the absolute deviation between each estimated standard deviation and its corresponding true value. The average of these absolute deviations ($\:\stackrel{-}{MAD}$, Eq. 2) across replicates was then used to evaluate which variability measure (SD_individual_ or SD_pooled_) more closely aligned with the true SDs in each scenario.

2 $$\:\stackrel{-}{MAD}=\frac{1}{N}\sum\:_{j=1}^{N}\left(\frac{1}{k}\sum\:_{i=1}^{k}\left|{\widehat{s}}_{i}^{\left(j\right)}-{s}_{i}\right|\right)$$

where $\:{\widehat{s}}_{i}^{\left(j\right)}$ is the estimated SD for group *i* in simulation *j*, $\:{s}_{i}$ is the true SD for group *i*, *k* is the number of groups, *N* is the number of simulations. $\:{\widehat{s}}_{i}^{\left(j\right)}$ is equal to $\:{\widehat{s}}_{pooled}^{\left(j\right)}$ for pooled estimate (same for all *i*) or is equal to $\:{\widehat{s}}_{i,\:individual}^{\left(j\right)}$ for group-specific estimates.

To quantify the Monte Carlo uncertainty associated with $\:\stackrel{-}{MAD}$, the Monte Carlo Standard Error (MCSE, Eq. 3) was computed as the standard error of the simulated MAD values across *R* Monte Carlo simulations (in this study *R* = 10,000).

3 $$\:MCSE\left(\stackrel{-}{MAD}\right)=\frac{{s}_{MAD}}{\sqrt{R}}=\sqrt{\frac{1}{R\left(R-1\right)}\sum\:_{r=1}^{R}{\left({MAD}_{r}-\stackrel{-}{MAD}\right)}^{2}}$$

where *s*_*MAD*_ is the sample standard deviation of the MAD values across replications, *MADr* is the Mean Absolute Deviation obtained in simulation *r*, $\:\stackrel{-}{MAD}$ is the average MAD across the *R* Monte Carlo replications. The MCSE therefore provides a measure of the precision of the estimated mean MAD due to Monte Carlo sampling variability and is reported in Supplementary Information.

In addition to reporting average MAD values, the percentage of simulations in which the pooled SD had a lower MAD than the individual SDs were computed (Supplementary Information). This provided a practical measure of how often pooled estimates were more accurate than individual ones under specific experimental conditions.

#### Large SD deviations

To identify substantial estimation errors, large SD deviations were defined as cases in which an observed SD differed markedly from its true value (specifically, falling below − 50% or above + 50% of the expected value). These deviations were then used to evaluate how well the estimated SDs reflected the true variability in each simulation scenario. Two metrics were computed for each simulation setup:

(i) individual-group deviation rate, representing the percentage of simulations in which an individual group’s SD estimate fell outside the ± 50% interval around its true value;
(ii) pooled-SD deviation rate, capturing the frequency with which the pooled SD estimate diverged by more than ± 50% from the population mean of the true SDs.

#### Use of Levene’s and F_max_ tests to support variability measure selection

Variance homogeneity tests are essential for determining whether parametric ANOVA can be applied to the raw data or transformations are required. Beyond their traditional inferential purpose of informing the applicability of parametric ANOVA, their outcomes (in terms of detected variance heterogeneity) can inform the selection of an appropriate measure of variability, as they are designed to identify non-homogeneous conditions.

To evaluate whether variance homogeneity tests can effectively support the choice between SD_individual_ and SD_pooled_, we applied Levene’s^21^ test and the F_max_ (Hartley’s) test^22^ across all simulated scenarios. We selected both tests, since, in the context of a meta-analysis, F_max_ could be applied *a posteriori* to estimate SDs from graph error bars without individual data, whereas Levene’s test would require access to the raw data. Note that Levene test is not applicable when there are fewer than three replicates. F_max_ tables do not provide critical values for *n* = 2. For these cases, we generated critical F_max_ values via Monte Carlo simulation (1 million simulations per value). We excluded the case *n* = 2 and k = 2, as critical values were not sufficiently reliable.

For each scenario, the outcome of the homogeneity test was used to guide the choice of the variability measure:

(i) if the test did not reject the null hypothesis of equal variances, SD_pooled_ was chosen;
(ii) if the test rejected the null hypothesis, SD_individual_ was used.

The performance of each test-guided strategy was assessed by calculating the MAD between the estimated and true SDs. A test decision was considered correct when the selected variability measure yielded the lower MAD relative to the true standard deviation values. The proportion of correct decisions across all simulations was then computed to quantify the effectiveness of each test in guiding SD selection.

## Results

### Distribution and bias of SD estimates in small to medium samples

**Fig. 2:**
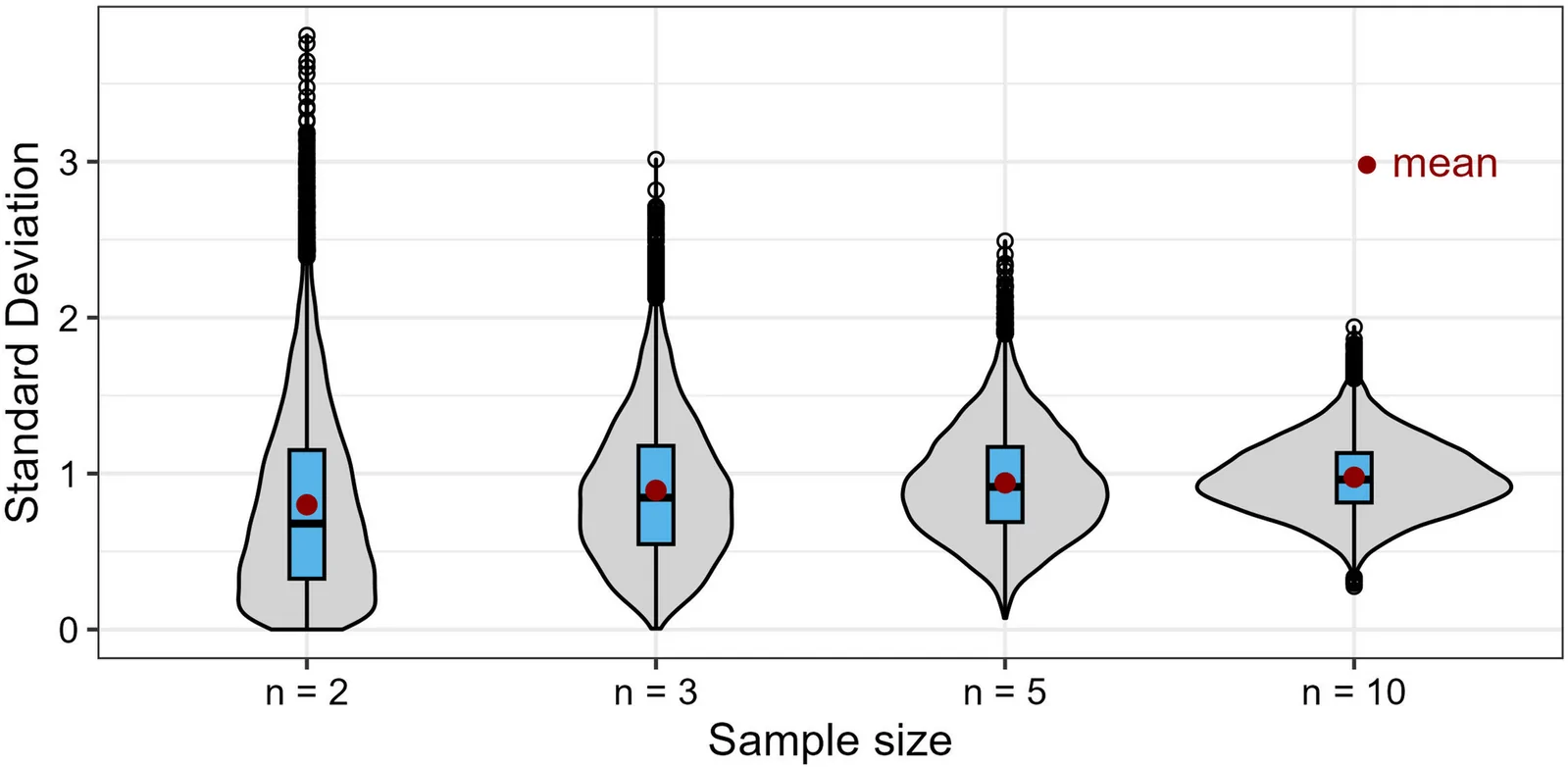
Combined violin and boxplots showing the distribution of simulated SDs for different sample sizes, assuming a true population SD of 1 consistent with previous findings reported in Kenney & Keeping^11^. Each distribution is based on 10,000 datasets for sample sizes 2, 3, 5, and 10. SDs mean values are reported as red dots.

To illustrate the behaviour of the sample SD in small samples, 10,000 normally distributed datasets were simulated for sample sizes of 2, 3, 5, and 10, assuming a population SD of 1. The resulting distributions^11^ were visualized using violin^23^ and boxplots to highlight how sample size influences the shape, spread, and bias of SD estimates (Fig. 2). The relationship between sample size and the distribution of SD estimates is well established in agreement with Kenney & Keeping^11^: smaller samples tend to underestimate variability and produce more dispersed estimates, while larger samples yield more stable and symmetric distributions around the true value. As shown in Fig. 2, increasing sample size reduces both the skewness and spread of the estimated standard deviations. For the smallest sample size (*n* = 2), the distribution is highly asymmetric, with a strong concentration of underestimated values, the interquartile range is larger, and the mean value has the greatest deviation and asymmetry observed for this sample size. As sample size increases, the distributions become progressively narrower and more symmetric, and the mean of the simulated SDs converges toward the true population SD. These well-known sampling patterns provide context for interpreting the subsequent analyses presented in this study.

### Assessing the accuracy of variability representation using MAD

**Fig. 3:**
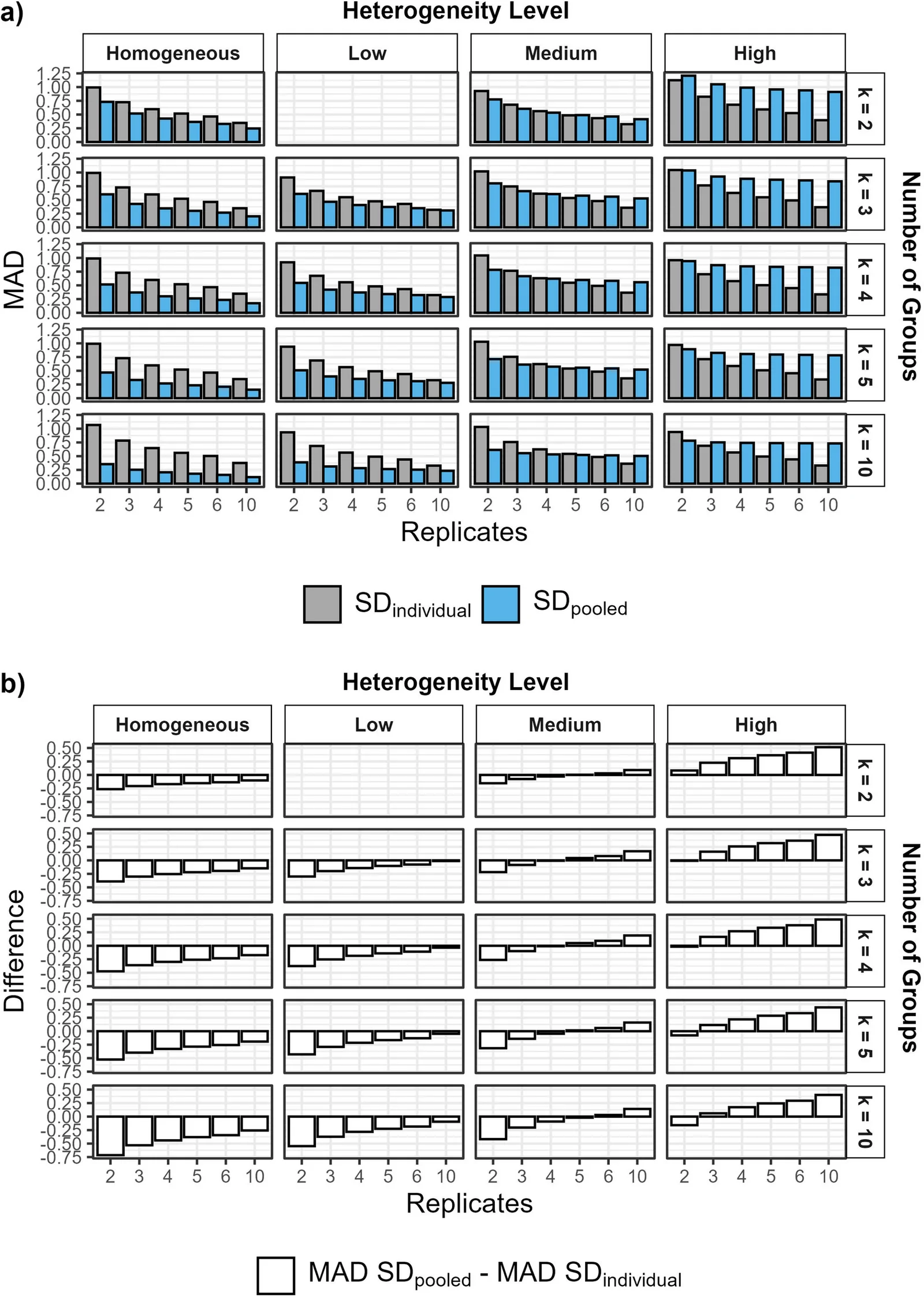
Mean Absolute Deviation (MAD) from the true population SD for pooled and individual SDs (**a**), and MAD difference between pooled and individual SDs (**b**) under combination of varying numbers of treatment groups (k) and variance heterogeneity levels. Positive values in (**b**) indicate superior performance of individual SDs, while negative values favour pooled SDs. Note that for k = 2, no “Low” heterogeneity bars are shown for either pooled or individual SDs. The Monte Carlo Standard Error (MCSE) associated with the MAD estimates is very small and therefore not visually discernible in the figure; full MCSE values are reported in the Supplementary Information.

Figure 3a displays the Mean Absolute Deviation (MAD) from the true population SD for pooled and individual SDs under the investigated combinations of replicates, treatment groups (k) and variance heterogeneity levels. The estimates of either SD_individual_ and SD_pooled_ were clearly affected by variance heterogeneity level and sample size, whereas the structure of the experiment, specifically the number of groups, has a less relevant effect (except for the high heterogeneity class when *n* = 2, Fig. 3a).

In the case of homogeneous variances, SD_pooled_ consistently exhibited superior performance, with markedly lower MAD values across all group sizes and replication levels (Fig. 3a). This advantage was particularly evident for very small samples (e.g., *n* = 2 or 3), where SD_individual_ showed greater instability and deviation from the true value. As replication increased, the difference between MADs for SD_individual_ and SD_pooled_ narrowed but remained in favour of the pooled approach.

Under low heterogeneity conditions (e.g., variance ratios of 1:2), SD_pooled_ continued to perform better, especially at lower replication levels. However, as heterogeneity increased (medium and high classes), SD_individual_ increasingly outperformed pooled estimates. This shift reflects the diminishing suitability of a single pooled measure to represent variability across groups when their true variances differ substantially.

This transition is summarized in Fig. 3b, which shows the difference in MAD between SD_pooled_ and SD_individual_. Negative values indicate scenarios where pooling reduces estimation error, whereas positive values identify conditions where individual SDs are more accurate.

### Large SD deviations of individual and pooled SDs

**Fig. 4:**
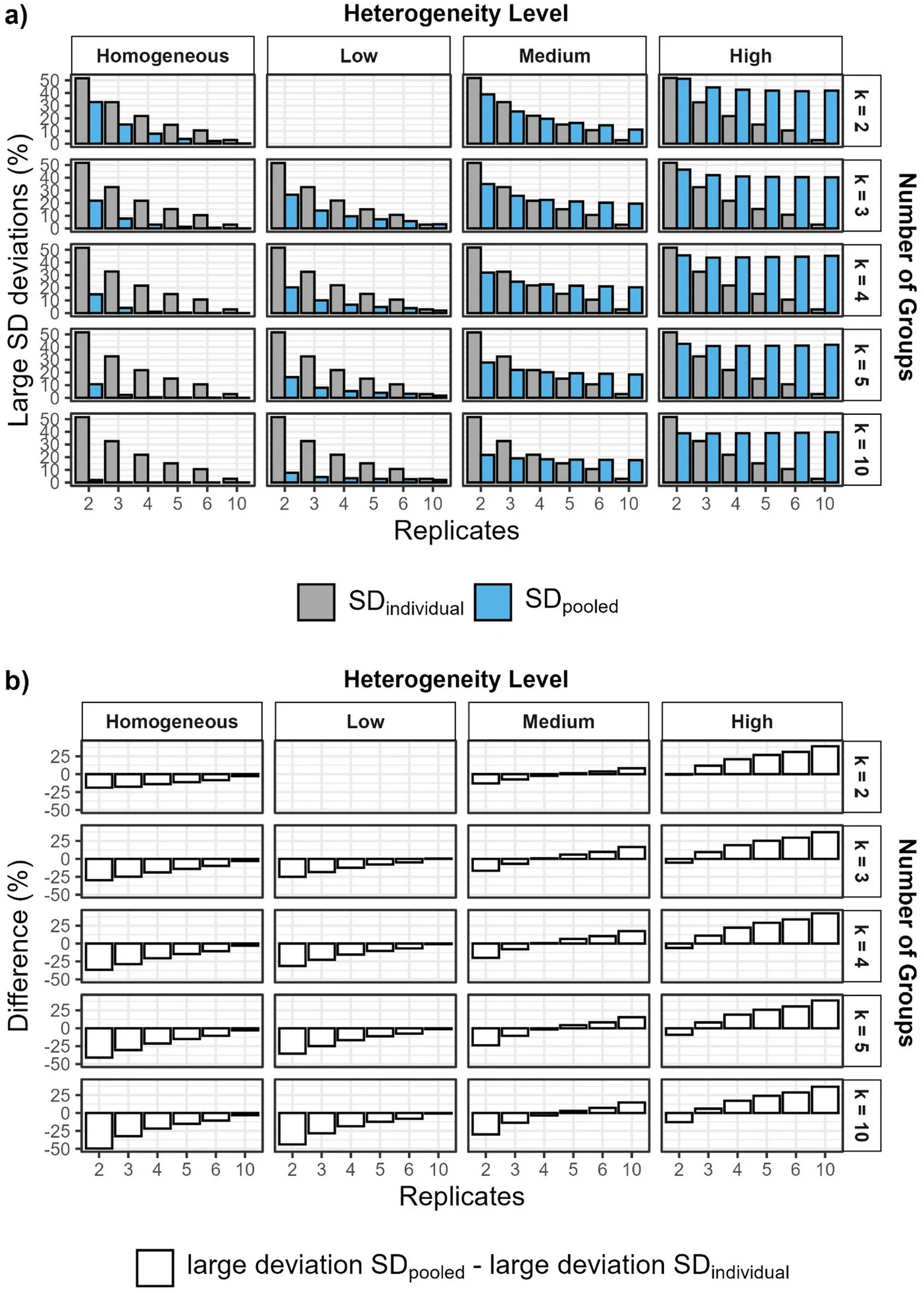
Percentage of simulations with large SD deviations (i.e., estimated SDs falling outside ± 50% of the true value) for pooled and individual SDs (**a**), and difference in large SD frequency between pooled and individual SDs (**b**) under combination of varying numbers of treatment groups (k) and variance heterogeneity levels. The values in (**a**) indicate the frequency with which each approach fails to approximate the true population variability. Positive values in (**b**) indicate superior performance of individual SDs, while negative values favour pooled SDs. Note that for k = 2, no “Low” heterogeneity bars are shown for either pooled or individual SDs.

Figure 4 reports the percentage of simulations with large SD deviations (i.e., estimated SDs falling outside ± 50% of the true value) for pooled and individual SDs under the investigated combinations of replicates, treatment groups (k) and variance heterogeneity levels. The percentage of simulations with large SD deviations (i.e., estimated SDs falling outside ± 50% of the true value, Fig. 4a) provided a complementary perspective on estimation robustness. Up to medium heterogeneity conditions (i.e., homogeneous, low, and medium heterogeneity), large deviations of SD_pooled_ were influenced by the sample size (decreasing for increasing n) while the number of groups had a minor effect. Under high heterogeneity, SD_pooled_ large deviations remained consistently high regardless of variations in k and n. In contrast, for SD_individual_ they decreased markedly with increasing sample size for all heterogeneity classes, regardless of k.

As expected, large deviations were most frequent when replication was limited (especially at *n* = 2 or 3) and occurred more often for SD_individual_. With only two replicates and high variance heterogeneity, more than 50% of the SD_individual_ exceeded the ± 50% deviation threshold. Pooled SDs performed substantially better under homogeneous or low heterogeneity conditions, particularly at small sample sizes, where the frequency of large deviations was generally below 10% in most cases.

However, the pooled approach performed poorly under high heterogeneity. In these scenarios, SD_pooled_ frequently underestimated the largest group variance, producing large errors in more than 40% of simulations. Conversely, SD_individual_ tended to overestimate variability in groups with low true variance, leading to distortions in relative comparisons across treatments.

Figure 4b reports for each investigated combination the difference in large SD frequency between pooled and individual SDs. It highlights the risk of misrepresentation inherent in both pooled and individual approaches, particularly in very small and small sample size experiments.

### Use of F_max_ and Levene’s tests to select a variability measure

**Fig. 5:**
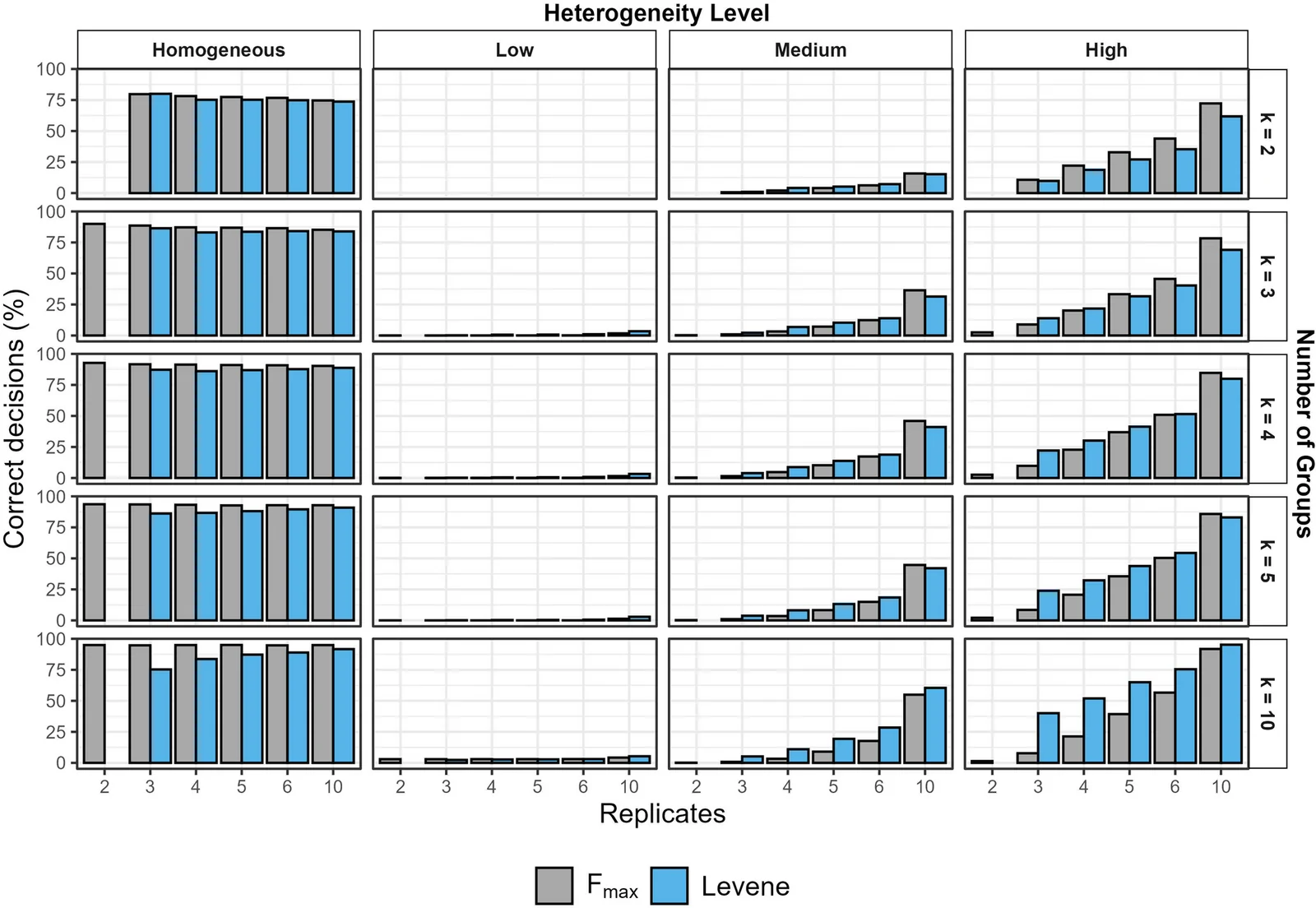
Percentage of correct decisions made by Levene’s test and F_max_ (Hartley’s test) at α = 0.05 under combination of varying numbers of treatment groups (k) and variance heterogeneity levels. The values indicate the percentage of simulations in which each test correctly identified the variability measure (pooled vs. individual SDs) associated with the lower Mean Absolute Deviation from the true SD values. Results are shown only for cases where each test is applicable (e.g., three or more replicates per group for Levene’s test).

Figure 5 shows the percentage of correct decisions made by Levene’s test and F_max_ (Hartley’s test) at α = 0.05 under the investigated combinations of replicates, treatment groups (k) and variance heterogeneity levels. In more than half of the experimental conditions considered, Levene’s test achieved a higher average percentage of correct decisions compared to the F_max_ test. Under low and medium heterogeneity (Fig. 5), Levene’s and F_max_ tests exhibited moderate decision accuracy, particularly when the number of replicates was below four. However, under non-homogeneous conditions, Levene’s test obtained better results in this experiment. In high heterogeneity scenarios, correct decision rates for some variance patterns exceeded 90% when the sample size was larger than six replicates. At moderate replication levels (*n* = 4 and 5), Levene’s test often led to the optimal decision for designs with four or more groups.

Concerning the performance of F_max_ test, accuracy was limited under low heterogeneity, particularly when the number of treatment groups exceeded two. Even with larger sample sizes (e.g., 10 replicates), the percentage of correct decisions generally remained below 70%, reflecting the test’s reduced sensitivity to subtle variance differences, especially when group variances are relatively similar.

As heterogeneity increased, the performance of the F_max_ test improved. In high heterogeneity scenarios with only two or three groups, it achieved higher decision accuracy when replication reached five or more (often exceeding 90% for some variance patterns). However, in more complex designs with 4, 5, or 10 groups, the test’s performance plateaued or declined, even under high heterogeneity and substantial replications.

### Selecting a variability measure in limited-size ANOVA experiments

**Fig. 6:**
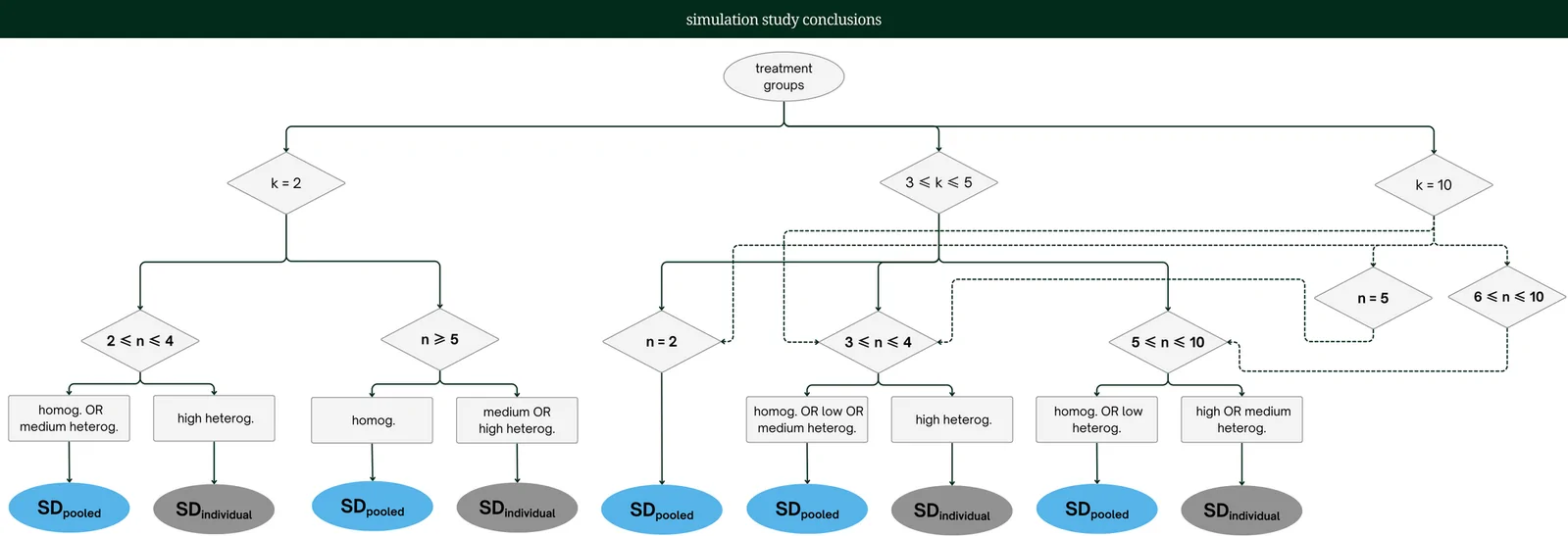
Decision tree to guide choice between pooled and individual standard deviations (SDs) based on the number of treatment groups (k), the number of replicates per treatment group (n), and the heterogeneity class.

The conclusions of the simulation study are summarized in a decision tree (Fig. 6, also available as an interactive Python or RStudio script with a console-based decision tool in the Supplementary Information). Decisions are made sequentially: first based on the number of groups, then on the number of replicates, and finally on the expected level of heterogeneity.

However, in many real-world experiments, researchers may not know in advance whether variances are equal across treatments. In such situations, especially in typical, balanced experimental designs where any variance heterogeneity, if present, is expected to be moderate (e.g., variance ratios up to 4:1), the following guidelines can help identify the most suitable variability measure.

If replication level is very low (very small sample size, *n* = 2 to 3): use SD_pooled_ by default. Even under moderate heterogeneity, SD_individual_ is too unstable to provide reliable estimates. The pooled SD minimizes random error and aligns with the ANOVA model assumptions. Levene’s test should not be relied upon in this case, as it is either not applicable or lacks statistical power with such small sample sizes.

If replication level is moderate (small sample size, *n* = 4 to 5): this represents a transitional zone. If there is no strong prior evidence of heterogeneity, SD_pooled_ is still a reasonable default, particularly with multiple groups. However, Levene’s test is recommended at this stage. If this test does not reject the homogeneity assumption at α = 0.05, continue using SD_pooled_. If the test does reject homogeneity, then switch to SD_individual_.

If replicates number is ≥ 6 (medium sample size): regardless of whether variance heterogeneity is suspected, the sample size is generally sufficient for Levene’s test to provide a reliable diagnostic. Follow the test result directly:

(i) if Levene’s test is not significant, use SD_pooled_;
(ii) if Levene’s test is significant, use SD_individual_.

## Discussion

This study set out to evaluate how best to represent variability in ANOVA experiments with small sample sizes, an issue frequently encountered yet often overlooked in agro-environmental research, and more generally in applied science.

Our findings confirm that SD estimates in small samples are both highly variable and systematically biased. As illustrated in Fig. 2, smaller sample sizes (particularly *n* = 2 or 3) produce highly skewed distributions of estimated SDs, often underestimating the true population value. This pattern is well-known from classical sampling theory and is a direct consequence of the shape of the chi-squared distribution underlying variance estimates^11,24^. In applied research, such underestimation may mislead readers into perceiving less variability than truly exists, especially when variability is represented using individual SDs derived from very small groups. 

The comparison of SD_pooled_ vs. SD_individual_ revealed that SD_pooled_ is generally more accurate (i.e., closer to the true values) under homogeneous or low heterogeneity conditions, especially when replication is limited. This advantage stems from the smoothing effect of pooling, which reduces random estimation error in small samples^25^. However, this benefit rapidly diminishes as heterogeneity increases. In those cases, SD_pooled_ conceals group-level variability and introduces systematic bias, particularly when higher variance groups are under-represented. In such contexts, SD_individual_ provides a more reliable representation of group-level heterogeneity, provided sample sizes are large enough to stabilize its estimates. These results reinforce the conditional validity of reporting SD_pooled_. While highly advantageous under homogeneity or low heterogeneity, particularly with few replicates, SD_pooled_ becomes misleading when substantial variance differences exist.

The analysis of large SD deviations reinforces this pattern for small sample-size ANOVA experiments. Pooled SDs suppress random noise and yield fewer large estimation errors when variances are homogeneous or nearly so. However, under heterogeneous conditions, pooled SDs risk concealing meaningful variability and introducing systematic bias. In contrast, SD_individual_ more accurately reflects true heterogeneity but can be volatile and noisy, especially when replication is limited. The practical takeaway of the simulated scenarios is clear: pooled SDs offer robustness, whereas individual SDs provide fidelity to group-level variability, each with trade-offs that depend on the underlying variance structure. The decision-support potential of variance homogeneity tests was also critically examined. Levene’s test showed generally higher decision accuracy than the F_max_ test in the investigated conditions, particularly in experiments with more than two groups or with moderate heterogeneity. This is consistent with its known robustness to departures from normality and its relatively high power across diverse data distributions^21,26,27^. In contrast, the F_max_ test^22^, which depends solely on the ratio of the largest to the smallest group variances, is more sensitive to extreme values. Although effective at detecting strong variance imbalances in simple two-group designs, its performance declines as the number of groups increases or when heterogeneity is moderate or distributed across multiple groups. Levene’s test provides a more reliable basis for decision-making across a wider range of experimental contexts. The presented results highlight the practical value of Levene’s test as a decision-support tool for selecting the appropriate variability measure in the framework of small sample-size ANOVA experiments. When replication is sufficient (*n* ≥ 5), it offers a reliable guide for choosing the SD estimation strategy. Our evaluation of test performance used a decision-focused criterion: correctness was defined not (only) by classical Type I and II error considerations, but by SD measure with the lowest MAD. This aligns more directly with the goal of data reporting (namely, to represent variability as accurately as possible) rather than with strict hypothesis testing. This approach also echoes recent calls to emphasise estimation accuracy and decision utility over reliance on p-values or binary test outcomes in statistical reporting^28,29^.

Taken together, these findings support a tiered framework that, by balancing estimation accuracy with robustness, provides a practical strategy for researchers working with small to moderate sample sizes (*n* ≤ 10). Focusing on estimation error (MAD) rather than exclusively on classical hypothesis tests yields actionable guidance for reporting variability in ANOVA designs, improving both clarity and credibility in small-sample studies and supporting better synthesis in meta-analyses. The observed patterns of SD behaviour appear to be reasonably robust across different error metrics, as consistent trends were obtained when considering both MAD and the occurrence of large SD deviations. Future research could extend the proposed approach by evaluating alternative performance metrics (e.g., mean squared error, relative bias) and by exploring a wider range of variance ratios and heteroscedasticity scenarios.

For small sample sizes, we recommend, as a descriptive approach, reporting variability using ± 1 SD rather than CI and explicitly labeling the chosen metric in figure captions and table headers. Confidence intervals are strongly influenced by sampling variability and inflated by the large *t*-distribution critical values associated with small samples (e.g., at α = 0.95 and *n* = 2, CI bounds are more than six times wider than the ± 1 SD interval, and remain wider until *n* = 6). In contrast, the standard deviation provides a more stable and interpretable metric for exploratory interpretation.

The proposed decision framework is applicable across many experimental settings in soil and agro-environmental sciences, medical and clinical research, animal and veterinary sciences, and food science, where the number of replicates and treatments is often limited. Several examples illustrate its use. In soil incubation or microcosm experiments, the number of treatments is usually small (k = 2–3) and replication is very limited (*n* = 2–3). In these conditions, SD_pooled_ is recommended. Individual SDs are extremely unstable and tend to underestimate variability, whereas SD_pooled_ provides a more reliable measure of within-treatment dispersion. In greenhouse or pot experiments, typical replication (*n* = 4–5) and moderate treatment number (k = 3–5) often lead to some heterogeneity due to uneven growth or environmental variation. In this case, Levene’s test should guide the choice. If homogeneity is not rejected, SD_pooled_ remains preferable. If heterogeneity is detected, SD_individual_ better reflects true treatment-level variability. In field experiments, replication is often small (*n* = 3–5), while treatments may be numerous (k = 4–10). Under these conditions, variability among plots or management factors (e.g., fertilization rates, irrigation regimes) commonly lead to heterogenous variances. Here, Levene’s test can reliably determine whether pooling is appropriate: when homogeneity holds, SD_pooled_ summarizes variability efficiently. When heterogeneity is detected, SD_individual_ should be reported to avoid masking meaningful differences in treatment response.

These examples show how integrating replication (n) and treatment structure (k) allows researchers to select a variability measure that balances statistical stability with reliable representation of experimental uncertainty. It should be noted that when heterogeneity is detected, alternative analytical procedures should be considered, e.g., Brown–Forsythe ANOVA, mixed models, and bootstrap methods. When alternative approaches are adopted, consistency with the chosen analytical framework supports the use of group-specific (SD_individual_) error bars. Overall, our investigation suggests that no single measure of variability is universally optimal. Instead, the choice should be guided jointly by variance structure and sample size. Pooled SDs are justified and effective when variances are homogeneous or nearly so, especially in very small or small experiments. When heterogeneity is suspected or confirmed (particularly using Levene’s test), SD_individual_ provides a more honest depiction of variability. These insights can also inform meta-analysts by indicating when pooled variances may be safely extracted from primary studies and when they may misrepresent underlying differences^30^.

## Conclusion

This study investigated the accuracy and robustness of alternative approaches for selecting a variability measure in ANOVA-based experiments with small sample sizes. Levene’s test emerged as a practical tool for selecting the appropriate variability measure when experiments include at least six replicates. Overall, our results show that in the investigated conditions, pooled SDs are generally more stable and accurate under conditions of variance homogeneity or low heterogeneity, particularly when replication is limited (*n* ≤ 4). In contrast, our simulations highlight that individual SDs become preferable when heterogeneity is present, and sample sizes are sufficiently large to stabilize group-level estimates (typically *n* ≥ 5).

## Supplementary Information

Below is the link to the electronic supplementary material.

Supplementary Material 1

Supplementary Material 2

Supplementary Material 3

Supplementary Material 4

Supplementary Material 5

Supplementary Material 6

## Data Availability

The dataset generated for the current study is available in the Supplementary Information section.
