# Supplementary material for: Guidelines for selecting variability measure in limited-size ANOVA experiments
Source: Sci Rep. 2026 Jun 8;16:26138. doi: 10.1038/s41598-026-54181-0 (PMC13490610; doi:10.1038/s41598-026-54181-0)
Supplement: Supplementary file 1 [file 41598_2026_54181_MOESM4_ESM.docx]

## Supplementary information

**Figure S1**. Procedure to systematically explore variance-heterogeneity patterns. For each group size, all unique configurations of group-level variances were generated using a combinatorial approach, enabling an exhaustive evaluation of possible variance patterns across groups.

**Figure S2**. Example of patterns generation and classification for the case of three groups (k = 3): the full set of 20 unique patterns comprises 4 homogeneous cases and 16 heterogeneous combinations.
